# Emerging Signal Regulating Potential of Genistein Against Alzheimer’s Disease: A Promising Molecule of Interest

**DOI:** 10.3389/fcell.2019.00197

**Published:** 2019-09-20

**Authors:** Md. Sahab Uddin, Md. Tanvir Kabir

**Affiliations:** ^1^Department of Pharmacy, Southeast University, Dhaka, Bangladesh; ^2^Pharmakon Neuroscience Research Network, Dhaka, Bangladesh; ^3^Department of Pharmacy, BRAC University, Dhaka, Bangladesh

**Keywords:** genistein, isoflavones, Alzheimer’s disease, amyloid plaques, neurofibrillary tangles

## Abstract

Alzheimer’s disease (AD) is a progressive, irreversible brain disorder characterized by pathological aggregation of the amyloid-β peptide (Aβ) and tau protein; both of these are toxic to neurons. Currently, natural products are regarded as an alternative approach to discover novel multipotent drugs against AD. Dietary soy isoflavone genistein is one of the examples of such agents that occurs naturally and is known to exert a number of beneficial health effects. It has been observed that genistein has the capacity to improve the impairments triggered by Aβ and also it possesses the antioxidant potential to scavenge the AD-mediated generation of free radicals. Furthermore, genistein can interact directly with the targeted signaling proteins and also can stabilize their activity to combat AD. In order to advance the development of AD treatment, a better comprehension of the direct interactions of target proteins and genistein might prove beneficial. Therefore, this article focuses on the therapeutic effects and molecular targets of genistein, which has been found to target directly the Aβ and tau to control the intracellular signaling pathways responsible for neurons death in the AD brain.

## Introduction

Alzheimer’s disease is one of the most common neurodegenerative diseases that represent 60–70% of dementia cases (Uddin et al., 2019a; Mathew et al., 2019). Currently, it is predicted that around 44 million individuals have Alzheimer’s or related dementia and this number is anticipated to over 135 million by 2050 (Uddin et al., 2016d; Hossain et al., 2019). The major neuropathological AD hallmarks involve proteinous aggregates found in the form of intracellular NFTs, containing hyperphosphorylated tau (Holtzman et al., 2011; Uddin et al., 2019c), and extracellular senile plaques, containing Aβ deposits of heterogeneous lengths (Selkoe, 1998; Uddin et al., 2018c). Aβ peptides are generated by sequential proteolytic processing of APP (Selkoe, 1998; Uddin et al., 2018c). In case of autosomal dominant forms of early onset AD, mutations in *APP*, presenilin-1 (*PSEN1*) or presenilin-2 (*PSEN2*) are accountable (Campion et al., 1999; Żekanowski et al., 2003; Uddin et al., 2016c). In fact, the basis for the amyloid cascade hypothesis is based on the findings that mutations of this three-gene (i.e., *APP*, *PSEN1*, and *PSEN1*) greatly change the metabolism of APP, preferring the generation of aggregation-prone Aβ species (Ricciarelli and Fedele, 2017; Uddin et al., 2018a). The neurotoxic Aβ peptides are mainly produced by cleavage of APP by β- and γ-secretases (MacLeod et al., 2015; Uddin and Amran, 2018). As a result of this event, alteration of neurotransmitter (Reinikainen et al., 1990; Francis, 2005), as well as synapse and neuronal loss in the hippocampus and the neocortex (Dheen et al., 2007; Devi et al., 2017) are the other hallmarks of this disease that are developed. At this moment, there is no available treatment to cure AD (Uddin and Asaduzzaman, 2016). Therefore, the exploration of plant-derived bioactive compounds has greatly led to the discovery of new drug candidates for AD (Uddin et al., 2016b; Rahman et al., 2017; Jakaria et al., 2019). The polyphenols are found to contain anti-AD activity (Queen and Tollefsbol, 2010; Uddin et al., 2018b). In addition, they are the most diverse and largest group of organic compounds among many natural products.

Isoflavones are polyphenolic secondary metabolites (Pandey and Rizvi, 2009) produced mainly from the members of the Leguminosae family (Forslund and Andersson, 2017). The major isoflavones found in soybean are genistein and daidzein (Abdallah et al., 2012). In order to improve the postmenopause symptoms, several soybean extracts including genistein and daidzein are used as a substitute for estrogen (Glazier and Bowman, 2001). It has been observed in rats that the soy isoflavones can protect against the Aβ_42_ peptide-mediated impairment of memory and learning (Xi et al., 2013). On the other hand, by means of the regulation of the nuclear factor erythroid 2-related factor 2 (Nrf2) signaling pathway, isoflavones were observed to avert the Aβ_42_–induced oxidative damage in the cerebrovascular tissue (Xi et al., 2014). Among different soy isoflavones, genistein constitutes about fifty percent of the total isoflavone content (Xu et al., 2015).

Genistein suppresses Alzheimer’s pathology by regulating copious intracellular events. In this article, we have primarily emphasized on the current understanding of the signal regulating potential of genistein to abate AD pathogenesis.

## Chemistry of Genistein

**FIGURE 1:**
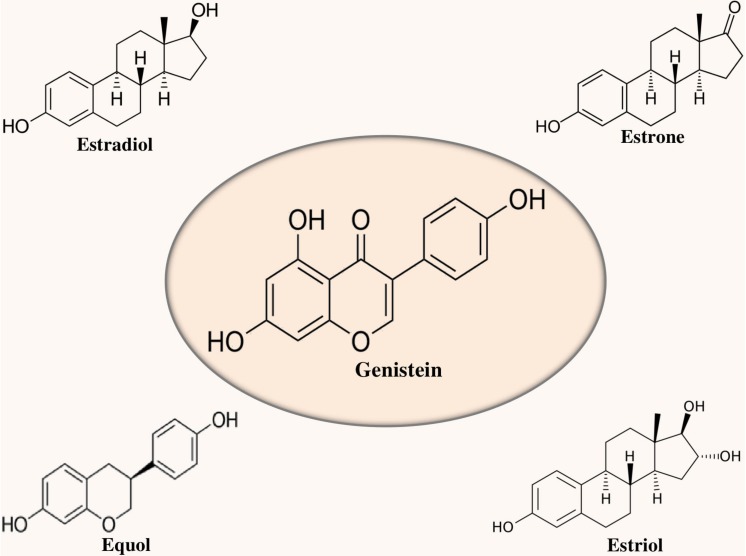
Chemical structure of genistein in relation to estrogens.

Genistein (4′,5,7-trihydroxyisoflavone) is a soy-derived isoflavone and phytoestrogen (Miadoková, 2009) as shown in Figure 1. It is regarded as a central intermediate which is required in the generation of more complex isoflavonoids (Dixon and Ferreira, 2002). Genistin (4′,5,7-trihydroxyisoflavone 7-glucoside) and its aglycone genistein (i.e., 4′,5,7-trihydroxyisoflavone) are the main isoflavones found in soybeans (Zubik and Meydani, 2003). In the plants, genistein is produced from the naringenin, an omnipresent flavanone. It has been observed that naringenin goes through the abstraction of hydrogen radical at C-3 afterward migration of B-ring from C-2 to C-3 and subsequent hydroxylation of resulting C-2 radial. In the presence of molecular oxygen and nicotinamide adenine dinucleotide phosphate (NADPH), the aforementioned reaction is catalyzed by isoflavone synthase, a stereoselective microsomal cytochrome P450 enzyme (Dixon and Ferreira, 2002).

Generally, acetylglucoside, malonylglucoside, aglycone, and glucoside are the four different forms of isoflavones that are found in soybeans (Xu et al., 2000; Zubik and Meydani, 2003). Interestingly, genistein is a relatively selective estrogen receptor (ER) β agonist and has structural similarity with estrogen (see Figure 1). ERβ is present in the brain areas linked with memory and learning (Jacome et al., 2010; Bean et al., 2014). Furthermore, genistein binding to ERβ might play a role in its neuroprotective action and also can ameliorate the memory deficit of AD (Bang et al., 2004).

## Bioavailability of Genistein

Several pharmacokinetic studies revealed poor oral bioavailability of genistein (Supko and Malspeis, 1995; Yang et al., 2012). However, consistency of genistein in terms of pharmacokinetic behaviors is still unclear because of its unpredictability and uncertain outcomes experienced via various biopharmaceutical investigations (Yang et al., 2012). Interestingly, while the glycosidic form has observed to be absorbed partially, it has been observed that the aglycon part exerts better bioavailability as compared to the glycoside (Piskula et al., 1999). In rats, it has been concluded by the bioavailability studies of genistein that plasma levels of portal vein help to bring about the understanding of the contribution of deglycosylation in the intestine and liver and uptake uniqueness of glycosylated flavonoids (Steensma et al., 2006). On the other hand, in soybean, genistein’s malonylglucoside is found to be thermolabile and degraded subsequently due to the cooking to non-acylated glucosides (Dixon and Ferreira, 2002). In addition, genistein is found to be transported across human intestinal epithelial cell monolayers (Dixon and Ferreira, 2002). It is recommended based on the metabolism and absorption rate of the ingested genistein and genistin that genistein has higher bioavailability as compared to genistin. However, human study showed that genistein is absorbed similarly to its glucoside (Janice et al., 2007; Devi et al., 2017).

In adults, genistein’s plasma half-life was evaluated from its plasma appearance and disappearance curves for the period of 7.9 h (Janice et al., 2007; Devi et al., 2017). Peak concentration was found to occur following 6–8 h of consumption and high steady-state plasma concentrations were observed due to the soy-containing diet consumption. Interestingly, it has been observed that up to 50–800 ng/mL of genistein and daidzein were released from the soy food which contains 50 mg/day of total isoflavones. On the other hand, about 1–5 μM of genistein were released in the blood of infants who ingested soy-based formula of 6–11 mg/kg/day of isoflavones (that contain 4–7 mg/kg/day of total genistein). Whereas, 0.5 μM of genistein was found to be released in the blood of adults who ingested soy products at the rate of approximately 1 mg/kg/day of total genistein (Setchell et al., 1998; Janice et al., 2007).

## Anti-Alzheimer’s *In Vitro* Studies of Genistein

Currently, the antioxidant and neuroprotective property of genistein is regarded as effective to treat AD.

### Upregulation of PKC Signaling

**FIGURE 2:**
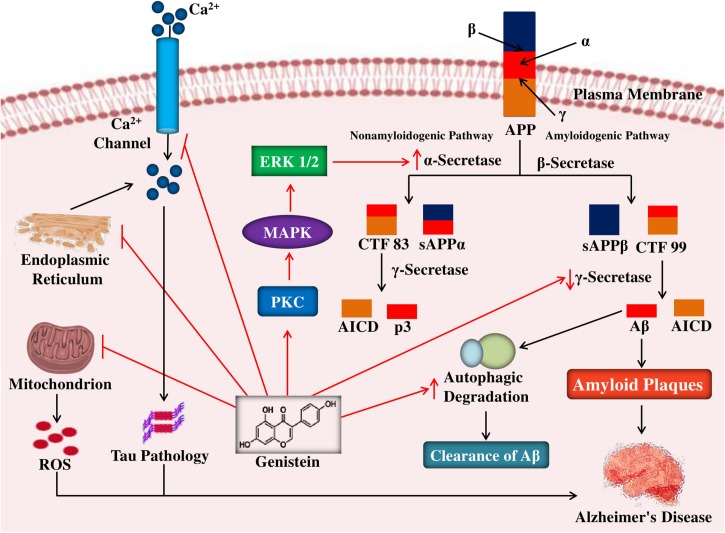
Mechanism of neuroprotective effects of genistein against neuropathological insults of Alzheimer’s disease. In the amyloidogenic pathway, cleavages of APP by β- and γ-secretases lead to the genesis of Aβ peptides and in the non-amyloidogenic pathway, cleavages of APP by α- and γ-secretases causes the formation of p3 and AICD. Genistein by activating PKC signaling pathway enhances the non-amyloidogenic pathway of APP cleavage by increasing α-secretase activity and in the amyloidogenic pathway genistein block the formation of Aβ peptides by reducing γ-secretase. Genistein also reduces tau pathology by blocking intracellular calcium levels as well as inhibit oxidative stress-mediated neuronal death by inhibiting the ROS release from the mitochondria as well as promote autophagic clearance of aggregate-prone proteins. APP, amyloid precursor protein; sAPP, soluble APP; CTF, C-terminal fragment; AICD, APP intracellular domain; ROS, reactive oxygen species.

Sequential processing of APP by β- and γ-secretases (Ortega et al., 2013) can cause the formation of neurotoxic Aβ, which can further accumulate as amyloid plaques (Uddin et al., 2019b; Harilal et al., 2019). In contrast, soluble APPα (i.e., non-toxic in nature) is formed due to the α-secretase mediated cleavage of APP (Adeniji et al., 2017). The role of α-secretase and reduction of the burden of amyloid plaque in the brain can be promoted by the enzyme protein kinase C (PKC, a phospholipid-dependent serine/threonine kinase). Therefore, agents that can cause inhibition of the enzymes β- and γ-secretases and activation of PKC could cause blockage of the amyloid plaque formation (Kim et al., 2011). In rat hippocampal neuronal cells, this positive action was noticed during treatment with genistein (i.e., 0.375 μg/mL), where it was observed that genistein protected the cells by a considerable increment of the α-secretase and attenuation of the β-secretase, via upregulation of the PKC signaling pathway (Liao et al., 2013) as given in Figure 2.

### Downregulation of Presenilin 1 and Ubiquilin 1 Expression

AD-linked ubiquilin-1 controls proteasomal degradation of proteins, comprising presenilin (Zhang and Saunders, 2009). Genistein can cause decreased Aβ generation by downregulation of the transmembrane protein presenilin which is found to be involved in the APP cleavage (Matsuda et al., 2011). On the other hand, in the lymphoid cells, genistein exerts an inhibitory action on the protein ubiquilin 1 which usually can cause stabilization of the presenilin (Matsuda et al., 2011). Though the molecular mechanisms by which ubiquilin 1 is controlled have not yet been recognized, this result may aid to develop defensive approaches against AD. In recent times, it has been stated that ubiquilin 1 controls proteasomal degradation of proteins comprising presenilin (Viswanathan et al., 2011).

### Reduction of Oxidative Stress

Oxidative stress (OS) is a central factor in the pathogenesis of AD (Uddin et al., 2016a). The generation of reactive oxygen species (ROS) in the brain can be increased due to the Aβ accumulation and can result in OS, which has found to cause apoptosis of neurons via the activation of caspases, cysteine-proteases, and DNA fragmentation (Radi et al., 2014). Fascinatingly, by reducing the ROS production, genistein (50 μM) exerts antioxidant effect against Aβ_25__–__35_ peptide-mediated OS in PC12 cells. It has also been found that genistein can mediate this aforementioned effect via inhibition of the mitochondrial permeability transition pore opening so that the ROS release from the mitochondria can be averted (Figure 2). The disturbed redox mechanism instigated by Aβ_25__–__35_ is eased by genistein primarily via the activation of nuclear factor erythroid 2/heme oxygenase-1 (Nrf2/HO-1) pathway (Ma et al., 2010).

Cerebrovascular disease (CVD) denotes a group of conditions that primarily disturb the blood supply to the brain (Hu et al., 2017). Although AD and CVD are not the same, it is often supposed that CVD coexists with AD (Love and Miners, 2016). It is recommended based on the evidence that Aβ deposition might be accelerated due to CVD and further can lead to AD pathogenesis (Crystal et al., 2014). On the other hand, it has been observed in several studies that due to the ability of Aβ to induce OS, it can change the capacity of cerebral endothelial cells to release the vascular relaxing factors and therefore can cause damage to the brain (Iadecola and Gorelick, 2003). Thus, it is expected that antioxidant therapy which can avert the Aβ-induced OS damage in the cerebrovascular endothelial cells might provide a protective action against AD. On the other hand, a study involving treatment of mouse brain endothelial bEnd.3 cells revealed that genistein (100 μM) can protect the cerebrovascular endothelial cells against the oxidative damage triggered by Aβ toxicity, by the maintenance of redox balance and ROS-scavenging via redox-sensitive signaling pathways. It is possible that the upregulation of phosphatidylinositol 3-kinase (PI3K)-mediated Nrf2 signaling might be the process through which genistein can provide protection to the cerebrovascular endothelial cells, as upregulation of PI3K and Nrf2, as well as further translocation of Nrf2 into the nucleus, were noticed in the endothelial cells (Xi et al., 2012).

### Suppression of Mitochondrial Damage

Mitochondrial abnormality is considered as a major pathological factor which usually takes place as an early AD event and the linked OS has found to cause neuronal cell death and degeneration. In case of sporadic AD, it has been found that Aβ deposition can take place because of mitochondrial dysfunction, this can further lead to the formation of NFTs and synaptic degeneration (Moreira et al., 2010). It was also observed that reduced glutathione/oxidized glutathione (GSH/GSSG) ratio, mitochondrial membrane potential, and mitochondrial membrane viscosity can be maintained by genistein. These aforesaid findings are based on the observed genistein’s (100 μM) effect on the Aβ-induced damage of mitochondria in PC12 cells.

On the other hand, one of the targets of genistein for prevention of mitochondrial membrane potential loss is by the prevention of mitochondrial permeability transition pore (mPTP) opening. It has been found that this opening mainly relies on certain stimuli for example depletion of adenosine triphosphate (ATP), overload of calcium, and ROS (Xi et al., 2011). The balance of redox is disrupted by the Aβ in the neurons; furthermore, Aβ can lead to mitochondrial abnormality via induction of DNA damage (Mao and Reddy, 2011; Ma et al., 2013). It has been observed that via GSH:GSSG ratio upregulation, Aβ-treated mitochondrial ROS accumulation can be inhibited by the treatment of genistein to the C6 rat glial cells. In addition, via the regulation of 8-oxoguanine glycosylase 1 (OGG1, an enzyme involved in the removal of 8-OHdG) expression and reduction of 8-Oxo-2′-deoxyguanosine (8-OHdG, an oxidized derivative of deoxyguanosine) levels, mitochondrial DNA damage in the cells can be prevented by the genistein (50 μM) treatment (Ma et al., 2013).

### Inhibition of Apoptosis

Genistein exerts neuroprotective effects to diverse kinds of cells against several toxic stimuli. It has been noticed that via ER activation, genistein abate the neuronal cell death (Linford and Dorsa, 2002). In a study by Zeng et al. (2004) reported that genistein ameliorates the cells from Aβ_25__–__35_-induced toxicity at a concentration of 0.1- and 40-μM in the neuronal cells of hippocampus collected from 1 day old neonatal Sprague-Dawley rat pups. Interestingly, it has been found that at both the concentrations, treatment with genistein caused a decrease in the production of ROS and fragmentation of DNA in the hippocampal cells, decreased the apoptotic indicator caspase-3 activation, and reduction in the number of apoptotic nuclear bodies and condensation of neuronal DNA (Zeng et al., 2004). It might be deemed that via the calcium level inhibition, genistein (see Figure 2) might possess an inhibitory action on the pathology of tau; since the induction of tau hyperphosphorylation via Aβ peptide is arbitrated via the enhanced level of calcium. Since at the physiological level (0.1 μM), the introduction of the antagonist of ER ICI 182,780 to the cells considerably blocked genistein’s effects, therefore prevention of programmed cell death in the cells by genistein is supposed to take place through an ER-dependent mechanism (Zeng et al., 2004).

It has found that Aβ_31__–__35_ peptides can cause induction of apoptosis in the cultured neurons of the rats via damaging the nuclear DNA integrity, reducing the mitochondrial membrane potential, and by further triggering bcl-2 downregulation and upregulation of p53, caspase, and bax (Yu et al., 2009). Fascinatingly, it has been observed that administration of genistein (100 μM) caused inhibition of apoptosis in the cells and also alleviation of caspase-3 upregulation, these phenomena provide further evidence for the role of genistein in the caspase-dependent pathway (Yu et al., 2009).

### Reduction of Aβ-Induced Neurotoxicity

One of the vital characteristics of AD is excessive neuronal cell death. Newborn Wistar rats were used to obtain primary neuronal cells in which genistein’s (100 μM) effect was evaluated against toxicity induced by Aβ_31__–__35_ peptide (Ding et al., 2011). Interestingly, the elevation of Aβ_31__–__35_ peptide-induced calcium overload and viscosity of the neuronal cells were found to be reversed due to the treatment of genistein. Elevated calcium overload and viscosity are the indicators of reduction in the fluidity of the neuronal membrane, it has been observed that genistein can reverse the Aβ_31__–__35_ peptide-induced fluidity alterations. Furthermore, ROS production can be reduced by genistein (see Figure 2); the overproduction of ROS is supposed to stimulate the signal transduction pathways which can cause cell death. Genistein was found to provide protection to the mitochondria of the cells through elevation of the mitochondrial membrane potential and reduced GSH:GSSG ratio, apart from showing general protective action in the neuronal cells. It has also been found that via inhibition of p38 MAPK and HCY-2, genistein may have a contribution in case of prevention of Aβ_31__–__35_ peptide-mediated toxicity, revealed via expression analysis of p38 mitogen-activated protein kinases (MAPK) and homocysteine-induced gene (*HCY-2*), a marker protein of apoptosis (Ding et al., 2011). Direct functional interactions between genistein and homocysteine are confirmed by *in vitro* experiment in order to determine the activities of glutathione peroxidase (GPx) and methylenetetrahydrofolate reductase (MetF), reconstructed with purified compounds based on measurement of the growth rate of the cultures of *Vibrio harveyi* and *Bacillus subtilis* (Banecka-Majkutewicz et al., 2017). The findings obtained from molecular modeling indicated that homocysteine has the ability to directly interact with genistein. It has been shown that homocysteine improved genistein-facilitated inhibition of MetF, on the other hand, genistein improved homocysteine-facilitated inhibition of GPx (Banecka-Majkutewicz et al., 2017).

Mitogen-activated protein kinases are protein Ser/Thr kinases that have a contribution in transferring the extracellular stimuli into specific cellular responses (Cargnello and Roux, 2011). It is known that p38MAPK is one of the subfamilies of MAPK. It has been noticed that upon upstream kinase phosphorylation it can become an active serine/threonine-protein kinase and found to cause further phosphorylation of a number of nuclear and cytoplasmic proteins. In case of AD, available evidence suggests that p38MAPK has a significant contribution. On the other hand, for treating AD individuals, inhibitors of p38 MAPK can be useful (Munoz and Ammit, 2010). Rat fetuses of fourteen to 15 days old were used to evaluate the action of genistein against alterations induced by Aβ in p38 MAP kinase. Here the primary cultures of rat cortical neurons were prepared from the cerebral cortex. Interestingly, it was noticed that Aβ_1__–__42_ toxic peptide-induced cell toxicity was prevented due to the treatment of the cells with 0.5 μM of genistein for the duration of 48 h. The ratio of GSSG/GSH and the levels of hydrogen peroxide and ROS were also found to be reduced by genistein. Furthermore, aggregation of mitochondria also found to be prevented by genistein, which otherwise can lead to cell death induced by caspase cascade. Through the prevention of p38 action, protection of neurons against Aβ-mediated neuronal cell death can also be achieved with the treatment of genistein, revealed by expression analysis of the neurons for p38 MAPK phosphorylation (Valles et al., 2010).

### Downregulation of the Neuroinflammatory Signaling

Neuroinflammation is mainly facilitated by microglia, shows a vital role in aging and neurodegeneration (Xia et al., 2018; Uddin et al., 2019d). In the brain of AD individuals, it was observed that glial cell activation could be caused by Aβ (Meda et al., 2001). Moreover, neuroinflammatory molecules can be secreted by these activated glial cells and can cause nerve cell damage, which further reveals that neuroinflammation is observed in the early stages of AD (White et al., 2005). On the other hand, it has been observed that pre-treatment with genistein can provide protection against the Aβ-produced inflammatory mediators [i.e., inducible nitric oxide synthase (iNOS), cyclooxygenase (COX), interleukin-1β (IL-1β), and tumor necrosis factor-α (TNF-α)]. These findings were obtained from the study involving observation of the genistein’s effect in Aβ (5 μM)-treated astrocytes. Moreover, Aβ-induced inflammation can also be prevented by the genistein (0.5 μM) and can lead to delay in the onset and advancement of AD. It is perceived that genistein-mediated activation of peroxisome proliferator-activated receptor-gamma (PPAR-γ) primarily cause this aforementioned effect (Valles et al., 2010). PPAR-γ has significant contribution in case of inflammatory response regulation; therefore anti-inflammatory property containing drugs, which has the ability to play a role as a PPAR-γ agonist, can cause suppression of the pro-inflammatory actions of Aβ in AD (Heneka et al., 2011).

Microglial cells are found to be activated via different stress signals and these microglial cells play a vital role in case of the inflammatory response (Dheen et al., 2007). In addition, microglial cells also contain toll-like receptors (TLRs), a specific type of pattern recognition receptors (PPRs) (Kielian, 2006). In humans, although several different functional TLRs have been recognized, among them TLR2 and TLR4 are regarded as most significant in terms of Aβ-mediated toxicity in AD individuals (Facci et al., 2015). It has been observed that in the treatment of the BV-2 cells with the aggregated Aβ_25__–__35_ forms caused enhancement of the expression of TLR2 as well as TLR4 and also induction of the inflammatory cytokine (i.e., IL-6) release. Genistein treatment (50 μM) inhibits the inflammatory action of Aβ_25__–__35_ by inducing downregulation of TLR expression. The aforesaid findings were based on the study involving the evaluation of genistein’s anti-inflammatory potential in the BV-1 cells (Yu et al., 2013).

In addition, it has been revealed in another study that via reduction of the expression of inflammatory mediators, for instance, iNOS and IL-1β; genistein (50 μM) can protect the BV-1 microglial cells against the Aβ_25__–__35_-induced inflammatory response (Zhou et al., 2014). It is believed that, in the microglial cells, the anti-inflammatory action of genistein is arbitrated through the reversal of TLR4 expression (i.e., which is upregulated by Aβ) and also found to be facilitated due to the downstream transcription of nuclear factor kappa-light-chain-enhancer of activated B cells (NF-κB). On the other hand, in the cytoplasm, this transcription factor usually occurs in the inactive form linked with regulatory proteins like p50 and p65. Interestingly, upon activation, the complex of NF-κB p65 p50 is found to translocate to the nucleus and can cause induction of the pro-inflammatory gene expression. It has also been found that via reversal of the Aβ-induced changes in the expression of NF-κB p50 and NF-κB p65, genistein can exert its anti-inflammatory action against Aβ. The action of genistein (50 μM) against neuroinflammation triggered by Aβ was studied in the C6 cells, in order to assess the genistein’s effect on the regulation of TLR4/NF-κB signaling pathway. Moreover, via suppression of the Aβ-induced inflammatory response, the C6 cells were observed to be protected due to the treatment of genistein. The aforesaid findings were based on several observations including a reduction in the levels of NF-κB, TLR4 downregulation, as well as upregulation of IL-1β, TNF-α, and IκB-α. In addition, through interfering with the TLR4/NF-κB signaling pathway, another study has shown that Aβ-induced neuroinflammation can also be prevented by genistein (Ma et al., 2015).

## Anti-Alzheimer’s *In Vivo* Studies of Genistein

Auspicious outcomes have been obtained by means of a number of pre-clinical assessments of genistein, which further recommends that genistein might be employed as potential drug therapy in case of AD treatment.

### Improvement of the Spatial Memory and Learning

Pan et al. (2000) revealed that memory could be improved through the oral intake of soy phytoestrogens to ovariectomized retired breeder rats. In an ovariectomized animal model of AD, the action of dietary soy meal ingestion (10 and 20 g) which comprised of with and without isoflavones (10 and 20 g) was assessed in order to evaluate whether postmenopausal dementia could be improved by the soy isoflavones. In the animals, learning and spatial memory were improved due to the feeding of soy meal, which further recommends that soya meal might be employed in case of AD treatment, as an alternative to estrogen (Sarkaki et al., 2008). In another study, rats were treated with a daily attainable high dose (10 mg/kg/day) and a low dose (1 mg/kg/day) of genistein. The findings revealed that a high dose of genistein can improve learning and spatial memory in rats. Nonetheless, treatment of genistein exerts no action on fear-driven learning and memory (Kohara et al., 2015). In AD animal model, the protective effect of genistein was evaluated against the damage of neurons induced by Aβ_1__–__40_ (Bagheri et al., 2011). This study suggested that genistein suggestively decreased the malondialdehyde content but did not show any effect on superoxide dismutase (SOD) activity and nitrite content. The outcomes recommended that pretreatment of genistein improves Aβ-mediated damage of short-term spatial memory in rats (Bagheri et al., 2011).

### Upregulation of Antioxidant Activity

**FIGURE 3:**
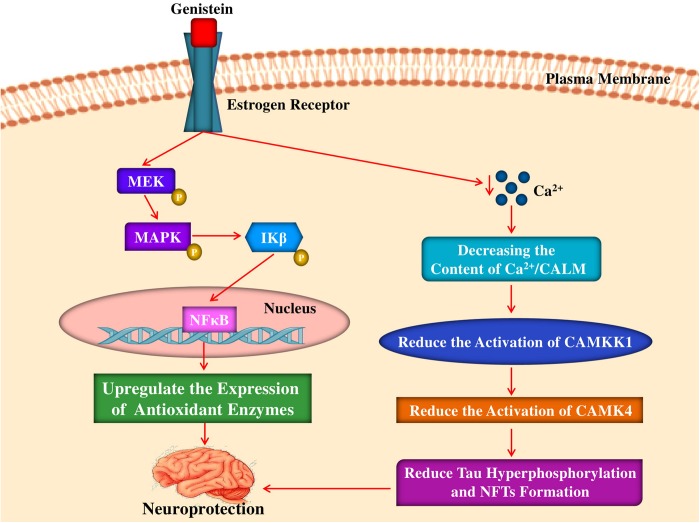
Mechanism of neuroprotective and antioxidant effects of genistein by interacting with estrogen receptor. Genistein binds to estrogen receptor activates a signaling pathway leading to phosphorylation of MEK, MAPK and subsequent phosphorylation of IKβ that causes translocation of NFκB and finally upregulating the expression of antioxidant enzymes. Genistein also abates intracellular calcium levels which leads to the reduction of CAMKK1, CAMK4 activation that leads to reduction of tau hyperphosphorylation and NFTs formation. MEK, mitogen-activated protein kinase kinase; MAPK, mitogen-activated protein kinase; IKβ, IKβ kinase; NFκB, nuclear factor kappa-light-chain-enhancer of activated B cells; CAMKK1, calcium/calmodulin-dependent protein kinase kinase I; CAMK4, calcium/calmodulin- dependent protein kinase IV; NFTs, neurofibrillary tangles.

Females live longer than males and the incidence of AD is higher in women as menopause may trigger AD (Janicki and Schupf, 2010; Viña and Lloret, 2010). Oestrogens protect females against aging by up-regulating the expression of antioxidant, longevity-related genes as well as mitochondrial toxicity of Aβ (Borrás et al., 2005; Viña and Lloret, 2010). Interestingly, Borrás et al. (2005) revealed the estrogen-mediated activation of NF-kB and MAPK directs expression of antioxidant enzymes, for example, manganese superoxide dismutase (Mn-SOD) and GPx. In a different study, the interaction of soy isoflavones with ERs was analyzed (Morito et al., 2001). It has been observed that binding affinities of equol, dihydrogenistein, and genistein are found to be comparable to the binding affinity of 17 beta-estradiol. In addition, the findings also revealed that genistin normally binds to the receptors more weakly and cause induction of transcription less than the genistein, also it can induce the growth of MCF-7 cells more strongly as compared to genistein. Mahn et al. (2005) reported that on long-term basis feeding of rats with a diet rich in soy protein during adult life and gestation can result in enhanced endothelial function, reduced OS, and decreased blood pressure in aged male offspring. On the other hand, enhanced vascular reactivity in animals fed a diet rich in soy protein was paralleled by elevated mitochondrial glutathione and mRNA levels for endothelial nitric oxide synthase (eNOS) and the antioxidant enzymes Mn-SOD and cytochrome c oxidase (Mahn et al., 2005). Interestingly, it has been noticed that genistein can play a role in a very similar fashion (Figure 3) to that of estradiol to the ER (Viña and Lloret, 2010). In a study, Ye et al. (2017) have revealed that genistein might have a neuroprotective function in AD via regulation of calcium/calmodulin-dependent protein kinase IV (CAMK4) in order to regulate hyperphosphorylation of tau. However, in the transgenic *Caenorhabditis elegans* CL4176 that expressing human Aβ_1__–__42_, treatment of genistein (100 μg/mL) was found to have no effect on the paralysis induced by Aβ (Gutierrez-Zepeda et al., 2005). In contrast, in the nematodes, another major isoflavone glycitein exhibited a protective action. It is conjectured that glycitein may suppress Aβ toxicity through combined antioxidative activity and inhibition of Aβ deposition (Gutierrez-Zepeda et al., 2005).

### Prevention of Aβ Aggregation

In the lateral blade of the dentate gyrus region of the brain, the formation of Aβ aggregation was prevented by the treatment of genistein (Bagheri et al., 2012). In addition to the aforesaid effect, treatment of genistein preserved this region which further recommends that, via an estrogenic pathway, genistein at a dose of 10 mg/kg has the capacity to avert the deficits of memory and learning in the rats injected with Aβ. The passive avoidance test and the Y maze test have revealed that the treatment of genistein can recall aspects of memory and learning and also enhance the short-term spatial recognition memory (Bagheri et al., 2012). It has also been revealed that that formation of Aβ aggregates can be prevented by the treatment of genistein to the animals (10 mg/kg) injected with the Aβ_1__–__40_ into the hippocampus of the rats.

### Regulation of BACE1 and NEP Actions

In a study, the action of genistein (26 μg/day) was assessed in a unique double transgenic/knockout APP 23 mice with an ovariectomy (APP/OVX; with a definite estrogen level in the brain) and with a genetic deficiency in aromatase (APP/Ar^+/–^; with non-detectable estrogen levels in the brain), which revealed a genetic deficiency of aromatase and decreased level of endogenous estrogen (Li et al., 2013). Since aromatase can lead to estrogen synthesis, therefore this enzyme is primarily targeted. Reduced levels of β-secretase, the formation of plaque, as well as Aβ_42_ and Aβ_40_ were observed in the genistein (26 μg/day)-treated APP/Ar^+/–^ female mice. On the other hand, neprilysin-2 (NEP2) is a protease which has found to cause Aβ degradation and can help in protecting against Alzheimer’s. Interestingly, it has been observed that treatment of genistein elevated NEP2 activity. As like estrogen, genistein might be more effective in case of AD prevention. The aforementioned phenomenon was observed when estrogen treatment was compared to the genistein’s effect. In order to halt the formation of amyloid plaque, study was also done to evaluate whether late and early treatment of genistein have an effect or not. In terms of early treatment, 3 months old female mice were subjected to genistein treatment and at 12 months of age, brain tissues were assessed to determine the occurrence of plaques. Furthermore, for both animals, treatment of genistein has decreased the number of plaques. In contrast, in terms of late treatment group, treatment of genistein was introduced at the age of 9 months, when the plaque has already found to be produced in the brain. Nonetheless, the formation of the plaque was not reduced by genistein. Henceforth, genistein treatment is found to be advantageous if it is introduced at an early age and this genistein treatment against AD relies on the estrogen level in the brain (Li et al., 2013).

### Activation of Aβ Clearance Pathway

Aβ aggregates are the foremost protagonist in AD (Oddo et al., 2006). In the CNS of a healthy human being, the Aβ production rate (i.e., 7.6%) is found to be lower as compared to the rate of Aβ clearance (i.e., 8.3%) (Bateman et al., 2006). Autophagy is a mechanism that involves vesicle and lysosome-arbitrated degradative process. Indeed, autophagy is vital for cellular health and protein homeostasis (Uddin et al., 2019a). In addition, autophagy is a vital regulator of clearance and generation of Aβ (Nilsson and Saido, 2014). Aβ clearance from the brain is another major target for anti-AD drugs in order to decrease neuronal death and synaptic defect (Lukiw, 2012; Uddin et al., 2019c). It has been revealed by Pierzynowska et al. (2019) that genistein can cause activation of autophagy at the higher dose (i.e., 150 mg/kg per day; this dose was markedly higher as compared to the doses used in previous AD studies) in a streptozotocin-induced rat model of the sporadic AD. In addition to this, they also observed that at this dose, genistein caused complete degradation of Aβ and hyperphosphorylated tau by stimulation of autophagy. Indeed, the behavior was found to be completely corrected (i.e., it was identical to healthy animals) in rats which were treated with a high dose of genistein. Furthermore, this observation was consistent in case of all the performed behavioral tests including open field test and locomotor measurements. It was concluded by the study that the autophagy-dependent process is accountable for genistein-facilitated reduction of AD pathology when this soy-derived isoflavone is particularly used at high dose (Pierzynowska et al., 2019).

Apolipoprotein E (ApoE) is another protein which is also regarded as a target for AD treatment since this protein is supposed to have a contribution in case of Aβ clearance from the brain (Ashraf and Uddin, 2019). Bonet-Costa et al. (2016) reported cognition and memory-enhancing capacity of genistein (0.022 mg/kg/day) in APP/presenilin 1 double transgenic (APP/PS1) AD mouse model. This aforementioned effect was linked with a reduction of Aβ levels in the brain, in the number and the area of amyloid plaques as well as in microglial reactivity. The primary reason for the aforesaid effect is genistein’s binding with the PPAR-γ moiety of the RXR (retinoid X receptor)/PPAR-γ dimer receptor. Moreover, from the astrocytes, ApoE release can be promoted by the genistein’s binding to the PPAR-γ, therefore recommending the uses of genistein for AD treatment (Bonet-Costa et al., 2016).

### Inhibition of Inflammatory Mediators

Various isoforms of the nitric oxide (NO) generating are raised in AD representing a role for NO in the pathogenesis (Lüth et al., 2002; Cau et al., 2012). In the animals, enhancement of the number of neuronal nitric oxide synthase (nNOS) expressing cells or enhancement of the intracellular nNOS expression may lead to increase in nNOS. In addition, in the genistein-treated animals, increase in nNOS may take place because of enhanced number of nNOS expressing cells, as genistein can lead to increase in the number of neurons in the DGlb area (Bagheri et al., 2012). However, treatment of genistein did not enhance expression of iNOS or the number of iNOS expressing cells, as aggregates of Aβ was found to have no action on the iNOS expression. It has been further revealed by glial fibrillary acidic protein (GFAP, a protein which is found in the glial cells) immunostaining that treatment of genistein can cause alleviation of astrogliosis mediated by Aβ_1__–__40_ injection into the hippocampal area of rats (Bagheri et al., 2012). Astrogliosis has significant contribution in case of inflammatory process noticed in AD. A study has assessed the morphological response of the astrocytes obtained from the Aβ_1__–__40_ and genistein (10 mg/kg)-treated animals. Generally, astrocyte activation can cause the increased generation of neurotoxic factors, for example, ROS, NO and cytokines which can ultimately lead to atrophy of the brain and neuronal cell death. In the animals, genistein treatment has found to improve the hypertrophy of the astrocytes triggered by the Aβ_1__–__40_ treatment in the animals (Bagheri et al., 2013).

## Clinical Studies of Genistein

In a study by File et al. (2001) reported that long-term and short-term memory could be considerably improved via the consumption of a high dietary soy diet (100 mg total isoflavones/day) in healthy young adults of both sexes for 10 weeks. Nonetheless, clinical studies regarding the therapeutic potential of genistein for AD treatment are still missing. Since copious promising pre-clinical data on genistein has been obtained, therefore further clinical studies can be done on genistein in the future. In addition, molecular interactions of genistein against the several other biomarkers of AD identified including complement receptor 1, clusterin, sphingolipids, and so on will further aid for better comprehension of the genistein’s disease-modifying action against AD (Devi et al., 2017).

## Conclusion

Several studies confirmed the neuroprotective actions of genistein on Aβ and tau peptide-triggered neuronal death. It has also been observed that genistein by activating PKC signaling pathway upregulates the α−secretase as well as downregulates β−secretase activities and in that way inhibits the formation of neurotoxic Aβ. Genistein could protect cerebrovascular oxidative damage by the activation of the Nrf2 signaling pathway by modulating PI3K activity. Genistein inhibits the ROS release from the mitochondria as well as block tau hyperphosphorylation by reducing intracellular calcium. Moreover, genistein can cause activation of autophagy to eliminate degradation of hyperphosphorylated tau and Aβ in the brain. Genistein could also bind to ER and causes upregulation of antioxidant enzymes and alleviation of tau pathology through regulating CAMK4. In fact, the neuroprotective effects of genistein are mediated by increasing antioxidant enzymes, reducing ROS, and suppression of mitochondrial toxicity, neuroinflammation, and apoptosis. Therefore, it can be summarized that genistein treatment might abate the pathogenic events of AD, which recommended further studies.

## Author Contributions

MU conceived the original idea and designed the outlines of the study. MU and MK carried out the work together, drafted the manuscript, prepared the figures, and read and approved the final submitted version of the manuscript.

## Conflict of Interest Statement

The authors declare that the research was conducted in the absence of any commercial or financial relationships that could be construed as a potential conflict of interest.

## Abbreviations

AD: Alzheimer’s disease
APP: amyloid precursor protein
A β: amyloid β
NFTs: neurofibrillary tangles.
